# Gastrointestinal Metastases From Primary Renal Cell Cancer: A Single Center Review

**DOI:** 10.3389/fonc.2021.644301

**Published:** 2021-03-23

**Authors:** Maelle Rony, Jean-Philippe Ratone, Jochen Walz, Geraldine Pignot, Fabrice Caillol, Christian Pesenti, Mathilde Guerin, Slimane Dermeche, Serge Brunelle, Naji Salem, Cecile Vicier, Stanislas Rybikowski, Thomas Maubon, Sami Fakhfakh, Manuel Tejeda, Marc Giovannini, Gwenaelle Gravis

**Affiliations:** ^1^Paoli-Calmettes Institute, Department of Gastrointestinal Disease, Marseille, France; ^2^Paoli-Calmettes Institute, Department of Urology, Marseille, France; ^3^Paoli-Calmettes Institute, Department of Medical Oncology, Marseille, France; ^4^Paoli-Calmettes Institute, Department of Radiology, Marseille, France; ^5^Paoli-Calmettes Institute, Department of Radiotherapy, Marseille, France; ^6^Paoli-Calmettes Institute, Department of Informatics, Marseille, France; ^7^Paoli-Calmettes Institute, Department of Medical Oncology, Aix-Marseille University, Inserm, CNRS, CRCM, Marseille, France

**Keywords:** metastatic renal cell cancer, gastrointestinal tract metastasis, incidence, treatment, digestive endoscopy, tyrosine kinase inhibitors, immune checkpoint inhibitors

## Abstract

**Introduction:** Digestive metastases (DMs) from renal cell cancer (RCC) are rare. Over the past decade, the overall survival of metastatic RCC (mRCC) has been improved by tyrosine kinase inhibitors (TKIs) and immune checkpoint inhibitors. The main objective of this study was to assess the incidence of metastases of the digestive tract in this new field of treatment. The secondary objectives were to evaluate the clinical characteristics, prognosis, treatments used for DMs, and median time between the diagnosis of RCC or mRCC and DMs.

**Materials and Methods:** A retrospective analysis of data collected from all patients with mRCC between 2007 (the time of TKI was a standard of care) and 2019 was carried out at the Paoli-Calmettes Institute (Marseille, France). Computer research software using artificial intelligence (ConSoRe®) was used to identify patients and assess their characteristics.

**Results:** Between January 2007 and December 2019, 11 out of 660 (1.6%) mRCC patients had metastases of the gastrointestinal tract. The median age was 62 years. Of the 11 patients, 81.8% experienced digestive bleeding or anemia. Only 2 patients were asymptomatic. The metastases were mainly duodenal (50%) and gastric (41.6%). The median time from cancer diagnosis and from metastatic disease to gastrointestinal metastasis was 4.3 years (3 months−19.2 years) and 2.25 years (0 days−10.2 years), respectively. Local treatment was performed in 38.5% of cases by endoscopy (60%), surgery (20%) and radiotherapy (40%) with success rates of 33, 100, and 50%, respectively. Etiological treatment was modified following the discovery of DM in 84.6% of the cases. The median survival was 1 year from the diagnosis of DM (13 days−9.4 years). Two patients were still alive 2.9 and 9.4 years after the diagnosis of DM.

**Conclusion:** This is the largest monocentric retrospective analysis of DM in patients with RCC. It seems to be a rare and late event in the course of the disease. Local treatment combined with systemic treatment could improve survival. In the context of prolonged survival with the new based immunotherapy treatments in mRCC, we suggest that unexplained anemia or persistent digestive symptoms could be explored by endoscopy.

## Introduction

Renal cell carcinoma (RCC) represents 3% of all cancers in France. Patients present with metastasis at the time of diagnosis in ~30% of cases (1). The lungs (45.2% of metastatic patients), bone (29.5% of metastatic patients), lymph nodes (21.8%) and liver (20.3%) represent the most common metastatic sites in clear cell renal cell carcinoma (ccRCC) (2).

Gastric metastases from ccRCC are very rare (3). The incidence of gastric metastases in different types of neoplasia ranges from 0.2 to 0.7% in autopsy studies (4–6), mainly in lung, breast, melanoma and esophageal cancer (7, 8).

Digestive metastasis (DM) from RCC is fairly poorly described in the literature and is generally the subject of case reports or literature reviews (3, 9). The management of metastatic RCC (mRCC) has evolved considerably over the past 15 years with the introduction of tyrosine kinase inhibitors (TKIs), and more recently, immunotherapy with immune checkpoint inhibitors, a combination of immunotherapy or combined with TKIs, has increased the lifespan of patients with metastatic clear cell renal cell cancer (mccRCC) (10).

We therefore carried out a retrospective monocentric study on the cases of DM of RCC over a given period from 2007 to 2019. The main objective of our study was to assess the incidence of DM in a population of patients with mRCC. The secondary objectives were to evaluate the clinical symptomatology, the characteristics of the DMs, the prognosis of the patients, the impact of systemic therapy (TKI and immune checkpoint inhibitors) used in these cases and the chronology of the appearance of DM in the history of cancer.

## Materials and Methods

### Study Design

This was a retrospective monocentric study (Institut Paoli-Calmettes, Marseille, France) enrolling patients over 18 years old with DM in a large cohort of patients with mRCC between January 2007 and December 2019. We chose 2007 as the date because of the marketing authorization of sunitinib, the first TKI indicated for mccRCC. DM could be located throughout the gastrointestinal tract (esophagus, stomach, duodenum, small intestine, colon or rectum). Only endoluminal metastases were considered. The exclusion criteria were pancreatic metastases, including pancreatic metastases with duodenal extension, locoregional digestive extensions of the renal tumor, and invasion of the gastrointestinal tract by peritoneal carcinosis.

Computer research software using artificial intelligence (ConSoRe®), developed in UNICANCER centers, was used to identify patients and assess their characteristics^1^. We reviewed the medical files of 139 patients found with the keywords “ICD C64—malignant neoplasm of kidney,” “with metastases,” and “digestive endoscopy” or “digestive hemorrhage” or “hematemesis” or “melena” or “rectorrhagia.” Eleven patients met the inclusion criteria. In this software, 660 patients presented with mRCC from January 2007–December 2019. The primary endpoint was the proportion of patients with DMs of mRCC in a population of patients with mRCC during the same time period.

The project was submitted to the internal protocol review committee of the Paoli-Calmettes Institute and was approved. Patients at the Paoli-Calmettes Institute are provided with general information regarding their right to object to the processing of their data.

### Statistical Analysis

All anonymized patient data were collected from the patient's computer medical file and entered into a spreadsheet. Continuous variables were expressed as medians with standard deviations. Eleven patients met the inclusion criteria for DM of mRCC, with one patient having presented 2 types of DMs (Table 1—Patient 11) and one patient with duodenal metastasis having been symptomatic 2 times in a 2.4 year period (Table 1—Patient 9). Therefore, we considered 12 metastases for the outcome “characteristics of the DMs” and 13 metastases for the outcome “treatment.”

**Table 1:** Population description.

		**RCC**		**Digestive metastases**				
**Patient**	**Age Sex**	**Pathology**	**Additional metastases**	**Main complaint**	**Details**	Interval from primary^*^	**Therapy**	Outcome^**^
1	57 M	ccRCC	Lung/nodes/bones	Melena	Two ulceroburgeoning lesions in the gastric small curvature and gastric antrum	4.3 years		Alive at 2.9 years
2	74 W	Papillary RCC	Nodes/bones	Hematemesis	Inflammatory bulb	3 months		Death at 19 days
3	73 M	ccRCC	Lung/bones	Anemia	Burgeoning lesion (50 mm) in the gastric body	10.2 years	Radiotherapy	Death at 18 days
4	77 M	ccRCC	Lung/liver/heart	0	Polypoid mass (30 mm) in the gastric fundus	4 years		Death at 3.5 months
5	56 W	ccRCC	Lung/bones	Anemia	Submucosal mass (25 mm) in the duodenum (D2)	1.25 years		Death at 13 days
6	74 M	ccRCC	Lung/nodes/ pancreas	Hematemesis	Infiltrating mass duodenum D2	19 years		Death at 9.4 months
7	61 M	ccRCC	Lung/liver	Melena	Atypical lesion (15 mm) in the duodenum (D2)	1.9 years	Endoscopy: clips, Hemospray® radiotherapy	Death at 1 years
8	54 M	ccRCC	Peritoneal carcinomatosis/ bones	Anemia/ rectorrhagia/melena	Endoluminal lesion (60 mm) in the jejunum	4 years	Surgery: proximal jejunum resected	Death at 2 years
9	62 M	ccRCC	Nodes/adrenal	Melena	Polypoid mass (35 mm) in the duodenum (D2)	10.2 years		Death at 4.2 years
	62 M	ccRCC	Nodes/adrenal/ liver/brain	Anemia/rectorrhagia 2.4 years after the first hemorrhage	Polypoid mass (35 mm) in the duodenum (D2)	10.2 years		Death at 4.2 years
10	58 M	ccRCC	Adrenals/bones	Anemia/melena	Gastric subcardial ulcer (20 mm)	6.3 years	Endoscopy: clips, Hemospray®, argon plasma coagulation	Death at 1.4 years
11	62 M	ccRCC	Lung/bones/ adrenals/pancreas	0	Nodule (15 mm) in the duodenum (D2)	5.6 years		Alive at 9.4 years
	69 M	ccRCC	Lung/bones/ adrenals/pancreas	Hematemesis/ rectorrhagia/ melena	Ulcerated lesions in the gastric body and fundus	12.4 years	Endoscopy: clips, Hemospray®	Alive at 2.6 years

*M, men; W, women; ccRCC, clear cell renal cell carcinoma*.

* *Interval from primary: time from diagnosis of renal cancer to diagnosis of DM*.

** *Outcome: time from diagnosis of DM to death or date of latest news*.

## Results

### Population

Between January 2007 and December 2019, 11 patients met the inclusion criteria for endoluminal DM of mRCC, namely, 8 men and 3 women (73% men, sex ratio 2.6). The median age was 62 years (range 54–77). The primary pathology was ccRCC for all patients except one who had papillary RCC (Table 1—Patient 2). All patients had other metastatic sites at the time of diagnosis of DM. Associated metastatic sites were the lung (63.6%), bone (63.6%), lymph nodes (36.4%), adrenals (27.3%) and liver (27.3%). Of the 11 patients in the study, 63.6% had 2 other associated metastatic sites, and 27.3% had 3 other associated sites.

### Primary Endpoint

The proportion of patients with DM in a population of patients with metastatic kidney cancer over the period 2007–2019 was 1.6% (11/660).

### Secondary Endpoint

#### Clinical Features

Of the 11 patients, 9 (81.8%) had clinical or biological symptoms related to DM. Only 2 patients remained asymptomatic (Table 1—Patient 4 and Patient 11). The symptoms found were gastrointestinal hemorrhage in 7 patients (melena, rectorrhagia, or hematemesis) or 63.6% of patients, and anemia was present in 10 patients (90.9%). Anemia was defined as a hemoglobin level <13 g/dL in men and <12 g/dL in women. The mean hemoglobin level at the diagnosis of DM was 9 g/dL with a median of 8.5 g/dL (6.5–13.9 g/dL).

Prior to the diagnosis of DM, anemia was present in 83.3% of cases 1 month before, 57.1% of cases 3 months before and 42.8% of cases 6 months before.

The discovery of DM was fortuitous in 3 patients. The gastric metastasis in Patient 4 (Table 1) was discovered during an echo-endoscopy to biopsy a suspicious hepatic nodule. Patient 11's duodenal metastasis (Table 1) was discovered incidentally on an evaluation CT scan and remained asymptomatic. Then, a gastric metastasis was diagnosed 7 years later on a digestive hemorrhage. Gastric metastasis in Patient 10 (Table 1) was initially diagnosed incidentally during esogastroduodenal endoscopy for recurrent abdominal pain in the context of pancreatic stent migration for chronic pancreatitis. Then, 8 months later, the patient presented with melena with anemia, and endoscopy revealed a known metastasis that was actively bleeding.

#### Characteristics of Digestive Metastases

For the statistical analysis of this section, we considered 12 metastases because one patient (Table 1—Patient 11) presented with duodenal metastasis and then gastric metastasis 7 years later. The tumor was localized in the duodenum in 6 cases (50%), in the stomach in 5 cases (41.6%), and in the jejunum in 1 case (8.3%). In the majority of cases, there was a single DM (8/12 or 66%).

The average size of the DMs was 31 mm. The type of lesion was extremely variable, as patients had submucosal, polypoid or ulcerated lesions.

#### Treatment

For the statistical analysis of this section, we considered 13 metastases, with one patient having presented 2 types of DMs (Table 1—Patient 11) and one patient with duodenal metastasis having been symptomatic 2 times in a 2.4-year period (Table 1—Patient 9). There was specific treatment in 5 cases (38.5%). Endoscopic treatment was used in 3 cases (60%), including hemostatic clips, hemostatic powder Hemospray® (COOK MEDICAL, Bloomington, Indiana, USA) and plasma argon, with a success rate of 33%. The treatment consisted of a surgical jejunal resection in one case (20%), with success. Hemostatic radiotherapy was used in 2 cases (40%) with a success rate of 50%. No patient benefited from vascular embolization.

DM required hospitalization for more than 1 day in 10 of the 13 metastases described in this section (76.9%), with a mean hospitalization of 11.4 days and a median of 11 days (2–24 days). Etiological treatment of kidney cancer was suspended for more than 4 weeks in 50% of cases. In all cases, the diagnosis of DM was made in the context of disease progression.

At the time of diagnosis of DM, systemic treatment was immunotherapy for 30.8% of patients, TKI for 46.1% and no systemic treatment for 23.1% of patients. The number of treatment lines prior to the diagnosis of DM was 0 (27.2%), 1 (27.2%), 2 (27.2%), or 3 (18.1%).

The etiologic treatment was modified following the discovery of gastrointestinal metastasis in 84.6% of cases. Four patients were started on nivolumab following the diagnosis of DM, with a median survival of 2.3 years from the diagnosis of DM (1.4–2.9 years).

#### Chronology

The median time from initial cancer diagnosis to the discovery of DM was 4.3 years (3 months−19.2 years). The median time to diagnosis of first metastases was 2.25 years (0 days−10.2 years).

The median time from symptom onset to the diagnosis of DM was 3 days (0 days−52 days). In 2 patients (Table 1—Patients 4 and 11), metastases were discovered incidentally and were therefore not included in this analysis.

#### Outcome

The median survival was 1 year from the diagnosis of DM (13 days−9.4 years). The times were highly variable, as 3 patients died <20 days after the diagnosis of DM, and 2 patients were still alive 2.9 and 9.4 years after the diagnosis of DM.

One of the patients (Table 1—Patient 1) is alive 2.9 years after the diagnosis of gastric metastasis discovered due to digestive hemorrhage. There has been no local treatment of the metastasis. He was on nivolumab at the time of diagnosis, and this treatment was not changed following the discovery of the lesion. The second patient (Table 1—Patient 11) is still alive at 9.4 years from the diagnosis of duodenal metastasis, which was initially discovered incidentally on a CT scan 5.6 years after the diagnosis of RCC. At 12.4 years after the initial diagnosis, he developed a digestive hemorrhage that revealed gastric metastases.

About glandular location metastases (pancreas, adrenal, thyroid, breast, parotid), 4 patients had glandular metastases (36.4%) at the time of the diagnosis of DM. The median survival in this group of patients was 32.8 months (9.2–112.3 months) vs. 3.5 months (13 days−34.4 months) in patients without glandular metastasis (HR 0.38; 95% CI = 0.09–1.5; *p* = 0.18).

## Discussion

DM of kidney cancer, whether gastric, duodenal or jejunal, is rare. In autoptic studies, the stomach has been reported as a metastatic site in 0.2–0.7% of cases, regardless of the primary site (4–6). The prevalence of RCC gastric metastases in the Pollheimer study was 0.2% over 22 years (3). Our study is, to the best of our knowledge, the largest monocentric study providing the prevalence of RCC metastasis throughout the digestive tract. Studies in the literature on RCC metastasis in the digestive tract are mainly case reports of 1 to 5 cases (11–23) or literature reviews (3, 9, 24–31). One of the limitations of this study is the single center design which limits the number of patients.

Like in our studies, the study by Kim et al., a literature review of 35 cases, found that those affected by DM were 74% men, with a median age of 62.4 years (range 54–77) (28). In the majority of studies, symptoms were mainly digestive hemorrhage (hematemesis, melena), anemia and abdominal pain. In our study, one patient presented with an incidental gastric metastasis during a diagnostic echoendoscopy to biopsy a liver lesion.

About localization, in the majority of cases, in the literature, gastric metastases of kidney cancer are located in the body of the stomach and are more often single lesions than multiple lesions (3). Colonic and rectal metastasis from several solid cancers have recently been described (32, 33). According to the 1947 study by Graham et al. of 195 cases of RCC, 4% of metastases were located in the small intestine (34), but metastasis bordering the primary tumor was also taken into account. Of all the segments of the digestive tract affected by RCC metastasis, the duodenum is the rarest (35). Duodenal metastases are most frequently located in the ampullary region (D2) and then in the bulb. Only ~20 cases of duodenal metastases of RCC have been described in the literature (21, 27). Lynch-Nyhan et al. described 3 cases (36) of duodenal metastases from RCC, 2 of which were treated by embolization of the gastroduodenal artery. On average, duodenal metastases appeared 7.9 ± 4.7 years after nephrectomy, and the median was 8 years (0–13 years) (27). One of the symptoms that we did not find in our study was jaundice when the lesion was ampullar (9). In several articles (34, 37, 38), pancreatic lesions invading the duodenum were described that were not included in our study. Thus, it is likely that lesions described as ampullary in the literature were classified in this category because of the difficulty, before jaundice, of distinguishing a duodenopancreatic mass visible on imaging and the modified endoscopic aspect of the papilla. There are 4 pathways of duodenal metastatic dissemination: peritoneal dissemination, direct contact with an intraabdominal tumor mass, and hematogenous or lymphatic pathways (9, 12). We considered only hematogenous or lymphatic diffusion, as described by Hashimoto et al. (11).

Anemia is very common in RCC, but it can get worse because of DM. We therefore suggest that endoscopic examinations be discussed at the onset or aggravation of, without clinical symptoms.

About jejunal metastases, they can appear many years after the initial diagnosis, and their diagnosis is difficult and requires extensive endoscopic explorations with enteroscopy, videocapsule endoscopy or exploratory surgery associated with enteroscopy (12). The combination of videocapsule endoscopy for screening and enteroscopy for histological samples seems to be a good approach to look for small intestine metastases in the context of digestive bleeding with normal upper and lower endoscopic explorations (18). Indeed, radical treatment combined with systemic treatment appears to improve survival (12, 39). In the case of massive digestive hemorrhage, the diagnosis can be made by angiography or during surgery.

DMs generally occur in the context of metastatic disease. In Pollheimer's review of the literature (3), 76% of patients with gastric metastases had other metastatic sites.

In the study by Gravis et al. (40), patients with mRCC with at least one glandular location (pancreas, adrenal, thyroid, breast, parotid), treated with antiangiogenic agents, were shown to have a longer overall survival time than patients without glandular location [median survival 61.5 months (51.4–81.6 months) vs. 37.4 months (31.3–42 months); hazard ratio (HR) 1.7; 95% confidence interval (CI) = 1.3–2.2; *p* < 0.001]. In our study, patients with glandular metastases had a longer median survival than patients without glandular metastases. Although this difference was not statistically significant, we can assume that this is related to a lack of enrollment and that there is still a trend. Therefore, the increased life expectancy of patients with TKI treatment and one or more glandular metastases could be a reason to discuss local treatment of DM in this subgroup.

The interval between diagnosis of the primary tumor and gastric metastasis depends on the primary tumor. For lung cancer and melanoma, metastasis is diagnosed an average of 2 years after the initial diagnosis (8). For RCC, the average interval is 7 years (0–24 years) (16, 26). In the Pollheimer et al. study of 17 patients, the mean interval was 6.5 years with a median of 4.7 years (0.1–20 years) (3), and the same timelines were found in Kim et al.'s study of 36 patients (28).

About the treatment, Cabezas-Camarero et al. have proposed recommendations for the therapeutic management of gastric metastases of RCC (24). Endoscopic mucosal resection or endoscopic submucosal dissection appears to have a place in the management of small, single lesions (28). Treatment with targeted therapy can also be considered, as shown in 2 cases treated with sunitinib and everolimus (14, 19), with nevertheless an increased risk of hemorrhage of the lesion. However, in the case of a large metastasis or active bleeding, local hemostatic treatment (by endoscopy or hemostasis radiotherapy) must be initiated before the introduction of targeted therapy (23). Many patients in the literature are treated by surgery (15, 16), but this must be carefully considered in view of the high morbidity (24) in patients with metastatic disease. Some authors recommend surgical resection of single and symptomatic gastric metastases (29).

The treatment of RCC with duodenal metastases depends on the stage of the tumor, the extent of the metastasis, and the characteristics of the patient. If the patient is eligible and there is no other metastatic location, surgery by cephalic duodenopancreatectomy may be considered. Otherwise, in the majority of cases, palliative care will be provided (21, 27).

The development of DM remains a poor prognostic factor representing a turning point in the disease. The median survival was 1 year from the diagnosis of DM but is still highly variable.

## Conclusion

DM in patients with RCC appears to be a late event in the course of the disease. In the majority of cases, they are discovered upon digestive bleeding, but sometimes only anemia is present. It is important to be aware that unexplained anemia or persistent digestive symptoms should be explored by endoscopy. However, due to the evolved molecular targeted and new immunotherapies, the survival period of the patients with mRCC was prolonged. Therefore, at the routine follow-up CT scan, we must be aware that formerly rare metastatic sites including digestive wall may increase. Local treatment by endoscopy, surgery or radiotherapy combined with systemic treatment could improve survival, especially in the era of immunotherapy in combination or associated with TKI.

## Data Availability Statement

The original contributions generated in the study are included in the article/supplementary material, further inquiries can be directed to the corresponding author.

## Ethics Statement

The studies involving human participants were reviewed and approved by Internal Protocol Review Committee of the Paoli-Calmettes Institute, Marseille, France. The patients/participants provided their written informed consent to participate in this study.

## Author Contributions

All authors participated in the writing of the article. All authors contributed to the article and approved the submitted version.

## Conflict of Interest

The authors declare that the research was conducted in the absence of any commercial or financial relationships that could be construed as a potential conflict of interest.

## Footnotes

^1^http://www.unicancer.fr/sites/default/files/big-data-analysis-clinical-records-paper-ConSoRe-white-paper.pdf
