# Corrigendum: Gastrointestinal Metastases From Primary Renal Cell Cancer: A Single Center Review

**DOI:** 10.3389/fonc.2021.710477

**Published:** 2021-06-14

**Authors:** Maelle Rony, Jean-Philippe Ratone, Jochen Walz, Geraldine Pignot, Fabrice Caillol, Christian Pesenti, Mathilde Guerin, Slimane Dermeche, Serge Brunelle, Naji Salem, Cecile Vicier, Stanislas Rybikowski, Thomas Maubon, Sami Fakhfakh, Manuel Tejeda, Marc Giovannini, Gwenaelle Gravis

**Affiliations:** ^1^ Paoli-Calmettes Institute, Department of Gastrointestinal Disease, Marseille, France; ^2^ Paoli-Calmettes Institute, Department of Urology, Marseille, France; ^3^ Paoli-Calmettes Institute, Department of Medical Oncology, Marseille, France; ^4^ Paoli-Calmettes Institute, Department of Radiology, Marseille, France; ^5^ Paoli-Calmettes Institute, Department of Radiotherapy, Marseille, France; ^6^ Paoli-Calmettes Institute, Department of Informatics, Marseille, France; ^7^ Paoli-Calmettes Institute, Department of Medical Oncology, Aix-Marseille University, Inserm, CNRS, CRCM, Marseille, France

**Keywords:** metastatic renal cell cancer, gastrointestinal tract metastasis, incidence, treatment, digestive endoscopy, tyrosine kinase inhibitors, immune checkpoint inhibitors

The authors names were incorrectly spelled as Rony Maelle, Ratone Jean-Philippe, Walz Jochen, Pignot Geraldine, Caillol Fabrice, Pesenti Christian, Guerin Mathilde, Dermeche Slimane, Brunelle Serge, Salem Naji, Vicier Cecile, Rybikowski Stanislas, Maubon Thomas, Fakhfakh Sami, Tejeda Manuel, Giovannini Marc and Gravis Gwenaelle. The correct spelling is Maelle Rony, Jean-Philippe Ratone,Jochen Walz, Geraldine Pignot, Fabrice Caillol, Christian Pesenti, Mathilde Guerin, Slimane Dermeche, Serge Brunelle, Naji Salem, Cecile Vicier, Stanislas Rybikowski, Thomas Maubon, Sami Fakhfakh, Manuel Tejeda, Marc Giovannini and Gwenaelle Gravis

The authors apologize for this error and state that this does not change the scientific conclusions of the article in any way. The original article has been updated.

